# Manipulation of the Host Cytoskeleton by Viruses: Insights and Mechanisms

**DOI:** 10.3390/v14071586

**Published:** 2022-07-21

**Authors:** Dahee Seo, Don B. Gammon

**Affiliations:** Department of Microbiology, University of Texas Southwestern Medical Center, Dallas, TX 75390, USA

The eukaryotic cytoskeleton comprises a network of actin, microtubules, and intermediate filaments that not only provide mechanical support to maintain cell morphology but also serve many other critical roles in cell motility, division, and intracellular transport of cargo such as vesicles and organelles. Recent evidence suggests that the cytoskeleton also participates in the regulation of host immune responses to infection by pathogens. These important roles of the host cytoskeletal network have made it a key target for manipulation by diverse DNA and RNA viruses.

Evidence for virus–cytoskeleton interactions were shown as early as the 1960s [1]. Since then, various components of the cytoskeleton network have been shown to be involved in virtually all steps of the viral life cycle. For example, numerous viruses have been found to re-organize actin structures near the plasma membrane to facilitate endocytosis-mediated viral entry [2,3,4]. Once inside the cell, many viruses hijack motor proteins on microtubules for transport to replication sites and for movement to the cell periphery for exit after replication [5,6,7]. At the same time, some viruses also modulate the expression of or re-organize cytoskeletal components to create an environment that favors viral replication [8,9]. As research on virus–cytoskeleton interactions progresses, it is becoming clear that unrelated viruses from different families have evolved both unique and common mechanisms to manipulate the host cytoskeleton in their favor, highlighting the importance of cytoskeletal machinery to viral infection.

Due to the importance of the cytoskeleton in the life cycle of virtually all viruses, it is critical to understand the mechanisms used by viruses to manipulate, usurp, and/or inhibit host cytoskeletal processes, as it may lead to new therapeutic strategies that can broadly target many important human viral pathogens. Furthermore, viruses can serve as excellent tools to study basic mechanisms of cytoskeletal network regulation and cytoskeleton-dependent processes. A deeper understanding of host cytoskeleton function may, in turn, lead to new therapeutics for diseases and pathologies resulting from cytoskeleton malfunction such as cancer and neurological disorders [10].

In this Special Issue, we publish papers with recent examples of how viruses that infect mammals, insects and plants manipulate cytoskeletal networks in their respective hosts. Khorramnejad et al. review the insect cytoskeleton in depth and show various examples of how viruses infecting insects utilize actin for short-distance transport while using microtubules for long-range transportation; they also describe how these transportation strategies contribute to horizontal and vertical viral transmission [11]. Zaghloul et al. additionally highlight how ascovirus infection alters the expression of cytoskeletal components in lepidopteran insect hosts to promote replication [12]. Another article in this Special Issue explores how Dengue virus infection and cytokine signaling synergistically contribute to changes in transendothelial permeability due to altered arrangements of the actin cytoskeleton, providing insight into underlying mechanisms of the increased vascular permeability typically associated with Dengue virus infection [13]. In addition, Seo and Gammon review the mechanisms employed by viral microtubule-associated proteins to alter viral transport, replication and immune evasion [14]. Overall, this Special Issue highlights the depth and breadth of cytoskeletal manipulations by viral pathogens that have been recently discovered and that have contributed to a greater understanding of virus–host cytoskeleton interactions.
